# *Silybum marianum* ethanolic extract: in vitro effects on protoscolices of *Echinococcus granulosus* G1 strain with emphasis on other Iranian medicinal plants

**DOI:** 10.1186/s41182-021-00363-7

**Published:** 2021-09-08

**Authors:** Ali Taghipour, Fatemeh Ghaffarifar, John Horton, Abdolhossein Dalimi, Zohreh Sharifi

**Affiliations:** 1 Department of Parasitology, Faculty of Medical Sciences, Tarbiat Modares University, Tehran, Iran; 2 Tropical Projects, Hitchin, UK; 3 Blood Transfusion Research Center, High Institute for Research and Education in Transfusion Medicine, Tehran, Iran

**Keywords:** Cystic echinococcosis, In vitro, Scolicide, *Silybum marianum*

## Abstract

**Background:**

Cystic echinococcosis (CE), is a parasitic zoonosis caused by *Echinococcus granulosus* (*E. granulosus*) larvae in liver and lungs of both humans and animals. Surgical intervention is the mainstay for CE treatment, using scolicidal agents that inactivate live protoscolices. This study evaluated the scolicidal effects of *Silybum marianum* ethanolic extract and its combination with albendazole in vitro for the first time. Moreover, in a literature review, we investigated the effects of a wide range of Iranian medicinal plants on protoscolices of *E. granulosus*.

**Methods:**

*S. marianum* ethanolic extract was prepared and high-performance liquid chromatography (HPLC) analysis was used to establish the proportions of its component compounds in the extract. Cytotoxicity was evaluated in mouse macrophage cells (J774A.1 cell line) using MTT method. Next, the scolicidal activity of the extract alone and combined with albendazole was tested as triplicate at various concentrations incubated for 5, 10, 20, 30, and 60 min. Finally, protoscolex viability was determined using 0.1% eosin as a vital stain. PCR–RFLP and DNA sequencing techniques were used to characterize the genotype of *E. granulosus*.

**Results:**

HPLC analysis showed that *S. marianum* ethanolic extract contained mostly silydianin (14.41%), isosilybin A (10.50%), and silychristin (10.46%). The greatest scolicidal effects were obtained with the combination of *S. marianum* with albendazole (79%), *S. marianum* ethanolic extract alone (77%) and albendazole (69%), at a concentration of 500 μg/ml for 60 min, respectively (*P* < 0.05). Molecular analysis showed that all the cysts used were G1 genotype.

**Conclusion:**

The data suggest that *S. marianum* ethanolic extract is a potential scolicide in vitro; however, further investigations are required to determine its efficacy in vivo.

## Introduction

The zoonotic tapeworm, *Echinococcus granulosus* (*E. granulosus*), is a genetically diverse metazoan parasite causing cystic echinococcosis (CE) or hydatid disease [1, 2]. Human CE infection has a global distribution with a resultant 1–3.6 million disability-adjusted life years (DALYs) worldwide; most of these cases occur in low- and middle-income countries [3, 4]. The predator–prey life cycle of *E. granulosus* consists of canids (definitive hosts) and herbivores/omnivores species (intermediate hosts) [5, 6], and human infection occurs accidentally by ingestion of *E. granulosus* eggs from soil, water and vegetables contaminated with infected canid feces [7, 8]. In humans, fluid-filled hydatid cysts are mainly found in the liver and lungs and, to a lesser extent, in the abdominal cavity, muscle, heart, bone and nervous system [9, 10]; as a result, it can cause a wide range of different symptoms and signs.

Currently, there are three treatment approaches for CE, surgical removal, PAIR (puncture, aspiration, injection, and re-aspiration), and chemotherapy using benzimidazole compounds [11–14]. Globally, surgery remains the most common treatment with chemotherapy as an adjuvant [15, 16], although this is not appropriate for all cyst stages [17]. Cyst rupture and spillage of cysts containing protoscolex-rich fluid during surgery is a significant cause of recurrence [18, 19]. Hence, the selection of a suitable drug to reduce recurrence is crucial [20].

Medicinal plants are considered as huge treasuries for a wide spectrum of valuable therapeutic compounds [21, 22]. *Silybum marianum* (Asteraceae), is a medically important plant species native to North and South America, Africa, Australia and the Middle East [23, 24]. The seeds of *S. marianum* contain a powerful substance called silymarin, which has long been used to support liver health [21]. Historically, therapists have used the seeds for their anti-carcinogenic and hepatoprotective effects [21, 25]. Therapeutic effects of *S. marianum* have also been demonstrated against prostate, skin and breast tumors, cirrhosis and kidney disease [26, 27]. Additionally, several studies have emphasized the anti-helminthic, anti-bacterial, anti-aflatoxin and immunomodulatory features of silymarin in *S. marianum* seeds [28–31].

Experimental studies of the scolicidal activity of ethanolic extract of *S. marianum* seeds are lacking; hence, the current in vitro investigation was designed to evaluate the scolicidal efficacy of *S. marianum* ethanolic extract alone or combined with albendazole. Also, in a literature review, we investigated the effects of a wide range of Iranian medicinal plants on protoscolices (PSCs) of *E. granulosus*.

## Materials and methods

### PSCs preparation and viability test

Livers and lungs of sheep infected with hydatid cysts were collected from the industrial slaughterhouse, Urmia, Northwestern Iran, and transferred, on ice, to the Parasitology Department, Faculty of Medical Sciences, Tarbiat Modares University at Tehran. Hydatid cyst fluid and PSCs were collected in sterile 50-mL tubes, then centrifuged at 2000 rpm for 5 min, as described by Smyth and Barret [32]. The supernatant was discarded and PSCs were washed four times in phosphate-buffered saline (PBS, pH: 7.2), supplemented with 0.5 mg/mL of amphotericin B, 200 mg/mL of streptomycin, 200 U/mL of penicillin and 10% glucose, in order to eliminate the rest of hydatid membranes and fluids [33]. Subsequently, 0.1% pepsin in Hank’s solution (pH: 2.0) was added to PSCs and incubated for 30–45 min to remove the remaining germinal layer and dead PSCs. Pepsin was removed by washing four times with Hank’s solution. To assess the viability of PSCs under light microscope, flame cell motility was examined using 0.1% eosin stain solution (1 g eosin powder dissolved in 1000 mL distilled water); PSCs, unstained after 5 min exposure, were considered as being viable [34]. PSCs with 99% viability were harvested and maintained at 4 °C for future experiments. PSC samples were also stored in 70% ethanol for molecular analysis.

### DNA extraction and polymerase chain reaction (PCR)

The genomic DNA of 50 μl of viable PSCs was extracted using a commercial DNA extraction kit, (Roche, Mannheim, Germany) according to the manufacturer’s instructions. Purified DNA samples were kept at -20 °C until further use. In the next step, PCR assay was set up to amplify a 462-bp segment of the ribosomal DNA internal transcribed spacer 1 (ITS1) gene and restriction fragment length polymorphism (RFLP) method was used for parasite genotyping [35]. Finally, PCR products were sequenced using Applied Biosystems 3730/3730xl DNA Analyzers (Bioneer, South Korea) and the results were compared using BLAST software against the GenBank database.

### Plant collection and extraction procedure

In the present investigation, *S. marianum* plants were collected from Tehran suburbs, followed by species authentication (herbarium number 514) by the Botany Division of the Iranian Biological Resource Center, Karaj, Iran. Plant seeds were ground to 0.4 mm diameter using a blender [36]. Next, 10 g of seed powder was extracted in a Soxhlet apparatus (Sigma- Aldrich), initially with 370 mL petroleum ether for 4 h, and then with 350 mL ethanol for 8 h. The ethanol was evaporated from the solution at 40 °C and an ethanolic extract of seeds (1.08 g) was obtained as a soft yellow powder (silymarin), which then dissolved in ethanol as stock solution. Experimental dilutions (500, 250, 125, 62.5, 31.25, 15.62, 7.81, 3.90, and 1.95 μg/ml) were finally prepared in Dulbecco’s modified Eagle medium (DMEM).

### High-performance liquid chromatography (HPLC) for silymarin content

HPLC is normally used to identify compounds present in plant extracts and compare their components. For this purpose, we used an Agilent 1290 Infinity LC System (USA). Extracts re-dissolved in ethanol and applied to a standard 250 mm × 4.6 mm HPLC column at a flow rate of 1 mL/min with the detector set at 288 nm for analysis of the chromatogram.

### Reference drug (albendazole) preparation and its combination with ethanolic extract of *S. marianum*

Albendazole (Sigma–Aldrich, St. Louis, USA) was dissolved in DMSO and used as a reference drug at concentrations of 500, 250, 125, 62.5, 31.25, 15.62, 7.81, 3.90, and 1.95 μg/ml, respectively. Combined concentrations for albendazole and ethanolic extract of *S. marianum* in equal proportions were 500, 250, 125, 62.5, 31.25, 15.62, 7.81, 3.90, and 1.95 μg/ml.

### Cytotoxicity assay by MTT

Mouse macrophage cell line (J774A.1), bought from Pasteur Institute of Iran, was cultured in cell culture flasks containing DMEM supplemented with 10% FBS at 37 °C, in a 5% carbon dioxide atmosphere [37]. 10^5^ macrophages were seeded into each well of 96-well culture plates and incubated for 24 h. Then, *S. marianum* concentrations were added to macrophages in different concentrations and the plates were incubated for 1 day at 37 °C. After addition of 20 μl of MTT solution (5 mg/ml), the plates were incubated at 37 °C for an additional 3–5 h, and the supernatant portion was discarded from each well. Finally, 100 μl DMSO was added to the wells, and 15 min later, optical absorption was read using an ELISA reader (Model 680, BIORAD Co.) at 570 nm. The cell survival was measured as follows:

Survival rate = (AT-AB)/(AC-AB) × 100; where AB is the optical absorption of the blank well, AC is the optical absorption of the control well, and AT is the optical absorption of the treated well.

### Analysis of scolicidal effects

For this purpose, 100/mL PSCs were added to DMEM containing 200 U/mL of penicillin, 200 mg/mL of streptomycin, and 0.5 mg/mL of amphotericin B in 96-well culture plates [38]. Subsequently, 0.5 mL of different concentrations of *S. marianum* extract and its combination with albendazole, were added to the respective wells, mixed gently and incubated for 5, 10, 20, 30, and 60 min at 37 °C. All experiments were done in triplicate. Occasionally, the number and viability of PSCs were determined using 0.1% eosin during incubation periods. The control for the study was non-treated parasites in DMEM. The PSCs fatality rate (PFR) was estimated using the following formula [39]:

$${\text{PFR}} = \frac{{\text{Number dead}}}{{{\text{Number live }}\left( {{\text{control}}} \right)}} \times 100$$

### Statistical analysis

ANOVA and t tests were used to determine the differences between tests and control groups; statistical analysis was performed using SPSS v. 17 (SPSS Inc., Chicago, IL, USA). A *P*-value of less than 0.05 was considered significant.

### Search strategy for literature review

For the literature review, national and international databases were searched using the following keywords: “Scolicidal agents”, “Medicinal plants AND *E*. *granulosus*”, “Herbal medicine AND *E*. *granulosus*”, “In vitro activity of plants AND Echinococcosis”, “Natural products AND Scolicidal”, and “Natural scolicidal”. Articles without full-text accessibility and those with confusing/unclear data and irrelevant papers were excluded.

## Results

### Genotype characterization

**Fig. 1:**
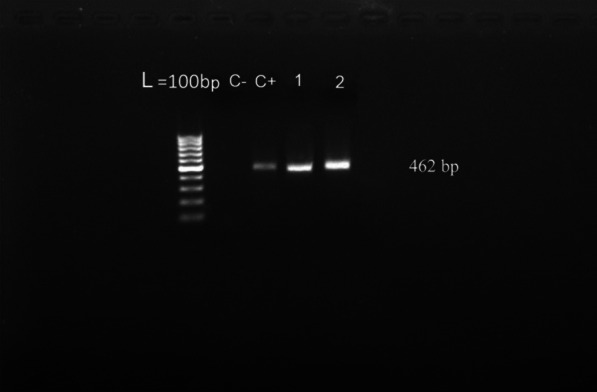
Electrophoresis of PCR amplification (462 bp) provided from liver of sheep sample (lane *C−* negative control, *C + * positive control, *lane 1 and 2* positive samples, *L* ladder 100 bp)

**Fig. 2:**
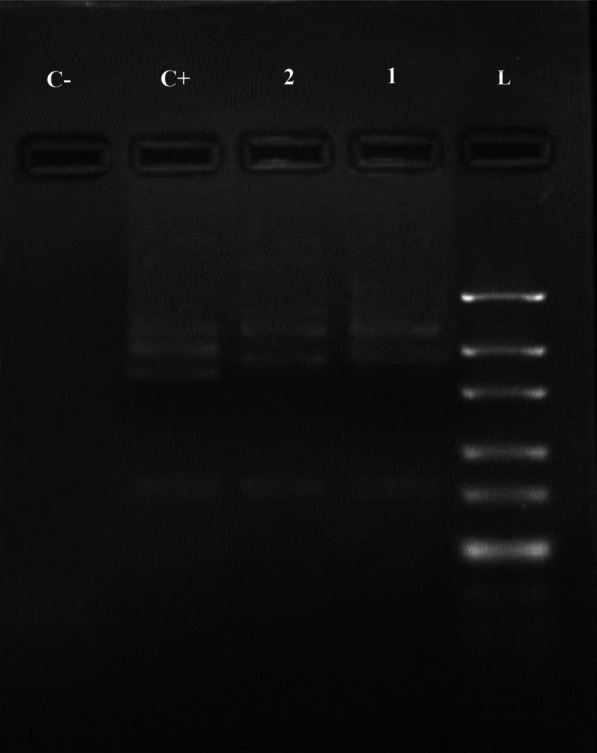
Agarose gel electrophoresis of ITS1-PCR products of *E. granulosus* isolates from sheep after digestion with the restriction enzyme Bsh1236I. (lane *C−* negative control, *C + * positive control, *lane 1 and 2* positive samples, *L* ladder 50 bp)

The amplified ITS-1 fragment showed a 462-bp band, as illustrated in Fig. 1. Subsequent PCR–RFLP analysis using Bsh1236I restriction enzyme demonstrated that isolated *E. granulosus* cysts were of G1 genotype (Fig. 2). A 99% homology was shown between the nucleotide sequences of the G1 isolate in current study and G1 registered in the GenBank database, under accession number MZ312242 and MZ312243.

### HPLC analysis

**Fig. 3:**
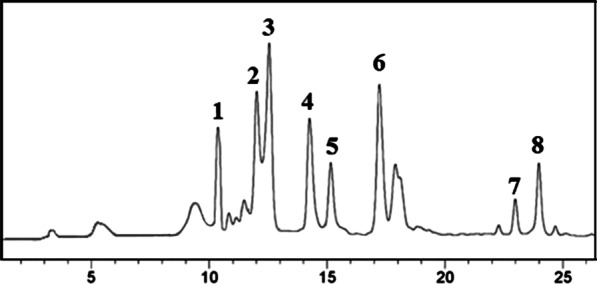
HPLC of silymarin constituents of the *Silybum marianum* ethanolic extract

As shown in Table 1 and Fig. 3, HPLC analysis of silymarin constituents of the *S. marianum* ethanolic extract revealed the presence of silydianin (14.41%), isosilybin A (10.50%), and silychristin (10.46%) at high level, while isosilybin B (3.04%) had the lowest relative concentration. All silymarin compounds are members of the flavonoid family.

**Table 1:** Percentage of compounds in ethanolic extract of *S. marianum* seeds obtained with HPLC analysis

Peak number	Compound	Percentage
1	Taxifolin	7.54
2	Silychristin	10.46
3	Silydianin	14.41
4	Silybin A	8.2
5	Silybin B	5.82
6	Isosilybin A	10.50
7	Isosilybin B	3.04
8	Isosilychristin	5.81

### Cytotoxicity assay

Over the range of concentrations tested, the greatest reduction (12%) in macrophage viability was seen at the highest dose (500 ug/ml *S. marianum* extract) (Table 2). Therefore, even at this concentration, the direct cellular toxicity of *S. marianum* extract was very low.

**Table 2:** Toxicity of ethanolic extract of *S. marianum* using MTT assay

Ethanolic extract of *S. marianum* concentrations (μg/mL)	Cell viability (%)
500	88
250	90
125	91
62.5	92
31.25	93
15.62	95
7.81	97
3.90	98
1.95	99

### Effect of S. marianum extract on PSCs

**Fig. 4:**
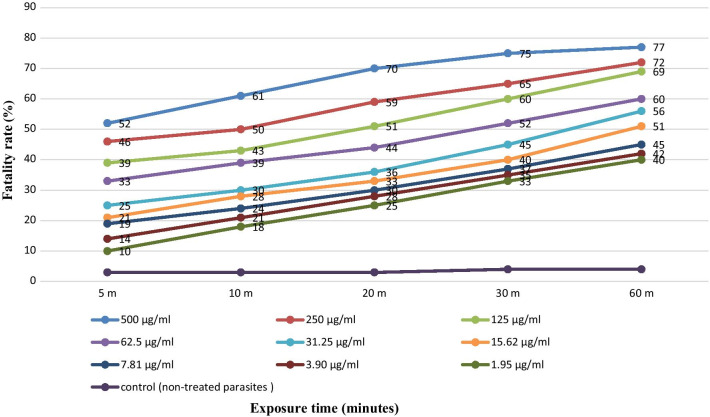
Scolicidal effects of ethanolic extract of *S. marianum* after exposure at different concentrations for 60 min. Each point represents the mean percentage of fatality rate

The results clearly showed that maximum effect was nearly 77%% with 500 μg/mL of ethanolic extract of *S. marianum* after 60 min (Fig. 4). Moreover, the scolicidal effects of *S. marianum* extract concentrations were significant in comparison to the controls in all exposure times (p < 0.05). It is noteworthy that most of the effect on PSCs was seen within in the first 5 min and showed a linear dose relationship. Beyond that time, there was approximately 30% additional effect over the subsequent 55 min for all concentrations.

### Effect of reference drug (albendazole) on PSCs

**Fig. 5:**
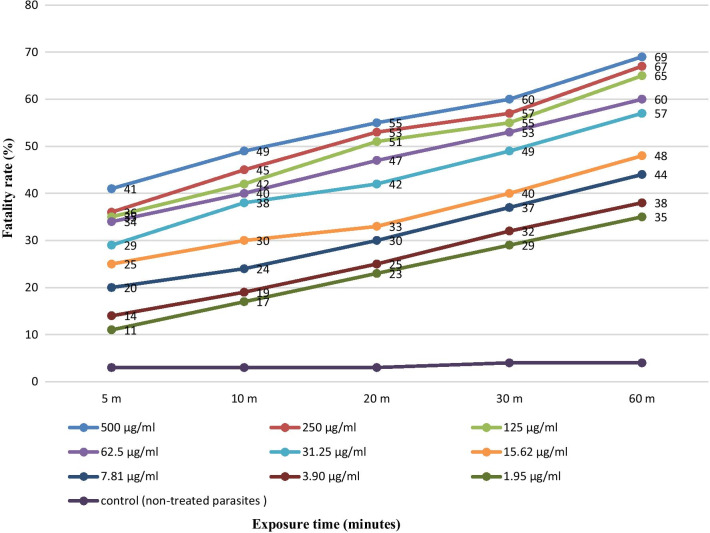
Scolicidal effects of albendazole after exposure at different concentrations for 60 min. Each point represents the mean percentage of fatality rate

The highest PFR was 69% at a concentration of 500 μg/ml after 60 min. The scolicidal effects of albendazole concentrations were significant in comparison with the controls at different exposure times (p < 0.05) (Fig. 5). The pattern of effects seen with the silymarin extract was also seen with albendazole.

### Effect of albendazole with S. marianum extract on PSCs

**Fig. 6:**
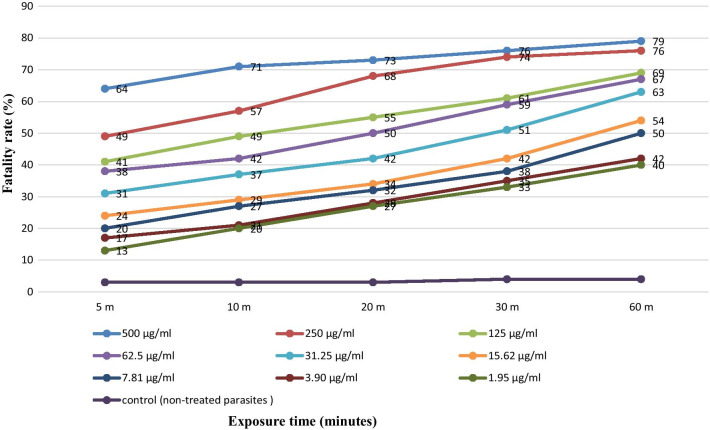
Scolicidal effects of ethanolic extract of *S. marianum* with albendazole after exposure at different concentrations for 60 min. Each point represents the mean percentage of fatality rate

The combination of albendazole and *S. marianum* extract was statistically significant at different concentrations, when compared with controls in 60 min (P < 0.05) (Fig. 6). The highest PFR with 79% was seen at a concentration of 500 μg/ml after 60 min. Again a similar pattern of effect, with the greatest impact within the first 5 min was seen with the combination. However, there is a suggestion that, at least at the highest doses the initial impact is greater than with the individual compounds.

## Discussion

Globally, surgery is considered to be the optimum approach for CE treatment [15] since the cysts are eliminated. However, anaphylaxis, disease relapse, recurrence or secondary dissemination due to the hydatid cyst fluid leakage may occur during surgery, presenting potentially lethal outcomes [40]. Surgery is also not an appropriate approach for certain cyst stages, very small cysts, multiple cysts and for locations other than the liver [17]. To overcome this problem, adjunctive chemotherapy can prevent or reduce recurrence (before surgery, after surgery, or both). In late 1970s, the first pharmacological compounds, the benzimidazole carbamates (mebendazole and albendazole) were introduced for human hydatid cyst treatment [41]. Numerous studies have shown that albendazole is significantly more effective than mebendazole in limiting and reducing the size of hydatid liver cysts [41, 42]. Today, it is the drug of choice for echinococcosis treatment being used before and after surgery or PAIR and for non-surgical intervention [42]. However, albendazole treatment, whether as an adjunct to surgery or as specific chemotherapy for situations where surgery is not appropriate, is not universally effective and is not always well tolerated [42].

In another in vitro study [43], the effect of albendazole (at lower concentration and for a shorter time) on scolicidal activity was greater than in the present study. There are some possible reasons for the difference in the effect of albendazole on PSCs, including drug resistance [44], type of albendazole synthesis [45, 46] and genetic structure and microRNA profile of helminth [47–49]. Some studies have suggested drug resistance of PSCs to albendazole [42, 44]. In this regard, one study showed that PSCs isolated from albendazole-treated gerbils became resistant to albendazole re-treatment after in vitro culture [44]. Therefore, it is possible that hydatid cysts isolated from infected sheep in the present study had a history of albendazole exposure and were thereby drug resistant. In future studies, it is suggested that a history of albendazole treatment be considered as an exclusion. On the other hand, some studies have shown that the scolicidal effects of the albendazole metabolite, albendazole sulfoxide, are better than albendazole [46, 50–52]. In the present study, we have used albendazole. More recently, some studies have suggested the presence of microRNAs of parasites as a factor in the resistance of various parasitic worms such as *E. granulosus* to anthelmintics [47, 53]. One study has shown that a high drug concentration or long-term exposure of the PSCs to albendazole resulted in high expression of miR-61 of *E. granulosus* compared to the control group [47]. The role of miR-61 in the growth, development and metabolism of different stages of *E. granulosus* is well defined [49, 54]. Therefore, further studies on microRNAs could be useful in understanding resistance mechanisms.

In this study, we defined the *E. granulosus* genotype since the effect of the compounds might vary depending on which of the 10 genetic genotypes (G1-G10) [55, 56]. Our results showed that all PSCs were G1 strain, which is the most common genotype found globally [55]. Although *E. granulosus* G1 strain is a major genotype in sheep, there have been reports of other genotypes such as G3, G6, and G7, in sheep [56]. To date, no studies have investigated the effect of different chemotherapeutic agents on the different *E. granulosus* genotypes. Therefore, it is suggested that PSCs of different genotypes (G1 to G10) be studied to gain a better understanding of the resistance or susceptibility of these genotypes.

In general, finding a suitable alternative drug has become an important challenge. Over the past 4 decades, various herbal extracts and chemical compounds have been investigated in vitro and in vivo as potential treatments [39, 57–60]. In the current study, an ethanolic extract of *S. marianum* and its combination with albendazole produced similar scolicidal activity compared to albendazole alone. Plants may induce or inhibit the activity of known anthelmintic agents due to their chemical content [61]. Increased albendazole anthelmintic activity may be due to the presence of secondary metabolites in the extract that facilitate drug uptake into the helminth tegument, thereby making drugs more available to the binding sites and ultimately enhancing the activity of the anthelmintic albendazole [61, 62]. Therefore, the compounds in the ethanolic extract of *S. marianum* may be a co-factor to enhance albendazole anthelmintic activity. Traditionally, *S. marianum* is an effective herbal product for a wide range of infectious and non-infectious diseases, with silymarin seen as the highly beneficial substance in its seeds [63, 64]. This compound contains silybin A, silybin B, isosilybin A, isosilybin B, silychristin, isosilychristin, silydianin as a mixture of polyphenolic substances as well as taxifolin as a flavonoid [65–67]. As shown in Table 1, silydianin had the highest concentration among the eight substances isolated. The proportions of the different compounds depend on the botanical origin of *S. marianum* in each country; silychristin (isolated from fruit), silybin A (isolated from stems), and silybin B (isolated from seeds) have the highest concentrations in Russia [68], Iraq [69], and Hungary [70], respectively. Several pharmacological outcomes might be expected with such a potent mixture of organic compounds, especially in fatty-liver disorders and non-alcoholic steatohepatitis [71, 72]. There is good evidence that silibinin is a supportive compound in recovery of Child–Pugh grade 'A' and alcoholic liver cirrhosis [73, 74]. Based on previous studies, *S. marianum* extract has also shown significant antimicrobial effects against fungi (i.e., dermatophytes and saprophytes) [75] and pathogenic bacteria (*Staphylococcus saprophyticus*, *Escherichia coli*, *Staphylococcus aureus* and *Klebsiella pneumoniae*) [76].

Iran has a great diversity in terms of medicinal plants. In our literature review, 25 studies were eligible for inclusion. As shown in Table 3, in addition to the current study 26 plant species from 23 genera and belonging to 12 families have been reported in the literature to have ethnomedicinal/ pharmacological properties as well as scolicidal activity against PSCs of *E*. *granulosus.* Although it seems that the in vitro studies of Iranian medicinal plants on PSCs are extensive, it is possible that useful data were missed within the ‘grey’ literature. Leaves were the most frequently plant part used (31% of studies). The scolicidal effects of medicinal plants range from 17.4% to 100% and the *S. marianum* extract used in the present study is comparable in activity to other Iranian medicinal plants investigated.

**Table 3:** Literature review of in vitro scolicidal effectiveness of various Iranian medicinal plants and their characteristics used against protoscoleces of *E*. *granulosus*

First author (reference)	Botanical family	Botanical name	Section used	Type of extract	Phytochemical components	Maximum scolicidal effects (%)	Concentration (mg/ml)	Exposure time (minutes)	Other reported therapeutic properties
Present study	Asteraceae	*Silybum marianum*	Seeds	Ethanolic	Taxifolin, silychristin, silydianin, silybin A, silybin B, isosilybin A, isosilybin B, isosilychristin	77	500 μg/ml or 0.5 mg/ml	60	Anti-microbial (helminthic, bacterial), anti-aflatoxin and immunomodulatory features Therapeutic effects of prostate, skin and breast tumors, cirrhosis and kidney disease
Faizei et al. [77]		*Artemisia aucheri*	Fruit	Methanolic	Linalool, borneol, decane, lavandulol	17.4	100	60	Anti-microbial (bacterial, leishmania, parasitic), anti-oxidant
Vakili et al. [78]		*Artemisia sieberi*	N/A	Aqueous	N/A	92.6	75	10	Anti-microbial (helminthic, bacterial, fungal), spasmolytic, anti-inflammatory, anti-oxidant, anti-tumors
Amiri et al. [79]		*Tripleurospermum disciforme* L	Leaves	Methanolic	N/A	100	50	10	Anti-bacterial, anti-inflammatory, anti-spasmodic, anti-septic
Gholami et al. [57]	Adoxaceae	*Sambucus ebulus*	Fruit	Methanolic	Flavonoids, steroids, tannins, caffeic acid, ebulitins, *α*- triterpenes	98.6	100	60	Anti-inflammatory, anti-nociceptive, anti-cancer, anti-angiogenic, anti-oxidative
Sadjjadi et al. [80]	Amaryllidaceae	*Allium sativum* L	Cloves	Aqueous	N/A	42.3	200	60	Anti-microbial (viral, bacterial, fungal, helminthic, protozoa), anti-tumor, anti-oxidant
				Hydro alcohol		71.4	200	60	
				Chloroform		99.5	200	30	
Moazeni & Nazer [81]				Methanolic	Alkaloids, saponin, flavonoids, steroids, cardenolides	100	50	10	
Eskandarian [82]				Chloroform	N/A	98	50	60	
				Hydro alcohol		92	50	60	
Rahimi-Esboei [83]			Flowers	Ultrasonic	N/A	98	100	180	
Haghani et al. [84]		*Allium cepa* L	Root	Methanolic	Tannins, flavonoids, alkaloids	21.8	100	60	Anti-microbial (bacterial, parasitic), anti-ascorbic
Mahmoudvand et al. [85]	Anacardiaceae	*Pistacia atlantica* Desf	Fruit	Methanolic	*β*-Myrcene, *α*-pinene, limonene	100	50	10	Anti-microbial, anti-inflammatory, anti-oxidant, anti-tumor, anti-asthmatic
Taran et al. [86]		*Pistacia khinjuk*	Leaves	Essential oil	Spathulenol, germacrene, *β*-pinene, myrcene, α- pinene	High	0.512	N/A	Anti-microbial (bacterial, viral) gastralgia, dyspepsia, peptic ulcer, anti-inflammatory, anti-pyretic
				Ethyl acetate		Low			
				Ethyl alcohol		Low			
				Chloroform	N/A	Low			
Mahmoudvand [87]		*Pistacia vera* L	Branch/ stems	Essential oil	Limonene, *α*-pinene, *α*-thujene, *α*-terpinolene, camphene, β-pinene	100	200	5	Anti-microbial (viral, bacterial, fungal, parasitic), anti-inflammatory, anti-nociceptive, anti-atherogenic, anti-diabetic
Moazeni & Mohseni [88]		*Rhus coriaria* L	Fruit	Methanolic	Tannins, flavonoids, terpenoids	100	50	10	Anti-bacterial, anti-oxidant, anti-fibrogenic, anti-diabetic, anti-tumorigenic, hypoglycemic
Mahmoudvand et al. [89]	Apiaceae	*Bunium persicum* (Boiss.)	Seeds	Essential oil	γ-Terpinene, cuminaldehyde, cuminyl alcohol, *ρ*-cymene, β-caryophyllene	100	25	5	Anti-microbial, anti-spasmodic, anti-oxidant, anti-inflammatory
Kavoosi et al. [90]		*Ferula assafoetida* L	Latex	Essential oil	α-Pinene, β-myrcene, decane	100	0.06	10	Anti-helminthic, analgesic, anti-septic, sedative, expectorant
Tabari et al. [91]		*Ferula gummosa* Boiss	Leaves	Essential oil	N/A	100	50 (ug/ml)	60	N/A
Lashkarizadeh et al. [92]		*Foeniculum vulgare* Mill	Seeds	Essential oil	trans-Anethole, α-pinene, limonene, methyl chavicol	100	1	5	Anti-oxidant, cytotoxic, anti-inflammatory, anti-mutagenic, anti-thrombotic, diuretic
Moazeni et al. [93]		*Trachyspermum ammi* L	Fruit	Essential oil	Thymol, γ-terpinene, *ρ*-cymene, linalool	100	5	60	Anti-helminthic, insecticidal
Rouhani et al. [94]	Berberidaceae	*Berberis vulgaris* L	Fruit	Aqueous	N/A	100	4	5	Anti-microbial (bacterial, parasitic, fungal)
				Hydro alcohol			2		
			Root	Methanolic	Isoquinoline alkaloid, carotenoid, flavonoid, tannin, flavonol, triterpene	100	2	10	
Eskandarian [82]	Betulaceae	*Corylus* spp	Seeds	Chloroform	N/A	36	50	60	N/A
				Hydro alcohol		33			
Sharifi-Rad et al. [95]	Brassicaceae	*Cardaria draba* (L.)	Leaves	Ethanolic	Isorhamnetin, quercetin, caffeic acid, sinapic acid, vanillin	67.6	10	60	Anti-microbial (bacterial, parasitic), anti-oxidant, anti-inflammatory
			Seeds		Caffeic acid, sinapic acid, quercetin, vanillin, isorhamnetin	66.3			
Bahrami et al. [96]		*Lepidium sativum* L	N/A	Essential oil	Thujene, myrcene, *ρ*-cymene	100	10	30	Dysentery, diarrhea, skin diseases, diuretic, leprosy, asthma
Eskandarian [82]	Cucurbitaceae	*Cucurbita* spp.	Edible part	Chloroform	N/A	47	50	60	N/A
				Hydro alcohol		44			
Tabari et al. [91]	Geraniaceae	*Pelargonium roseum*	Leaves	Essential oil	N/A	100	0.05	60	Anti-trichomonal and insect repellence
Taran et al. [86]	Lamiaceae	*Hymenocarter longiflorus*	Leaves	Essential oil	α-Terpinene, linalool, *ρ*-cymene, trans- caryophyllene	Methanolic and essential oil extract showed significant scolicidal efficacy with LC50 values of 135.88 and 79.68 μm/ml, respectively	N/A	N/A	Anti-microbial, anti-mosquito agent, larvicidal
			Stems	Methanolic					
Haghani et al. [84]		*Ocimum bacilicum*	Leaves	Methanolic	N/A	24.1	100	60	Anti-microbial (bacterial, fungal) and in treatment of splenomegaly
Zibaei et al. [97]		*Satureja khuzestanica*	Leaves	Ethanolic	Thymol, carvacrol, thymol acetate, γ-terpinene, *ρ* cymene, α-terpinene	100	1	30	Anti-microbial (bacterial, fungal, viral) Anti-diarrheal, anti-spasmodic, anti-inflammatory, vasodilator, anti-oxidant
Moazeni et al. [98]			Leaves	Essential oil	N/A	100	10	10	
			Flowers						
Kavoosi et al. [90]		*Zataria multiflora* Boiss	Leaves	Essential oil	Thymol, carvacrol, β-caryophyllene, linalool, α-terpinolene, *ρ*- cymene, thymol acetate	100	0.02	10	Anti-microbial (bacterial, fungal, protozoal), Analgesic, anti-septic, anti-inflammatory, anti-oxidant, immunostimulant, pain-relieving
Jahanbakhsh et al. [99]						100	12.5	5	
Zibaei [100]	Ephedraceae	*Ephedra major*	Roots	Methanolic	N/A	66.39 ± 34.61	0.1	60	Anti-bacterial, treatment of allergies, asthma, chills, coughs
			Stems			99.09 ± 2.27			
			Leaves			90.25 ± 19.42			

## Conclusion

In summary, an ethanolic extract of *S. marianum* is a potential alternative scolicide during PAIR treatment of cysts, and to reduce the risk from hydatid cyst fluid PSCs leakage during surgery and the subsequent complications. In the future, more research is needed to evaluate the in vivo effects of this group of compounds in a clinical setting in animal models and ultimately in humans.

## Data Availability

All data that support the findings of this study are available in manuscript text, Tables and Figures.

## Abbreviations

CE: Cystic echinococcosis
HPLC: High-performance liquid chromatography
PSCs: Protoscolices
DMEM: Dulbecco’s modified Eagle medium
ITS1: Internal transcribed spacer 1
RFLP: Restriction fragment length polymorphism
PAIR: Puncture, aspiration, injection, and re-aspiration
PFR: Protoscolices fatality rate
DALYs: Disability-adjusted life years
